# Differential Role of Circulating microRNAs to Track Progression and Pre-Symptomatic Stage of Chronic Heart Failure: A Pilot Study

**DOI:** 10.3390/biomedicines8120597

**Published:** 2020-12-11

**Authors:** Yuri D’Alessandra, Mattia Chiesa, Maria Cristina Carena, Antonio Paolo Beltrami, Paola Rizzo, Marta Buzzetti, Veronica Ricci, Roberto Ferrari, Alessandro Fucili, Ugolino Livi, Aneta Aleksova, Giulio Pompilio, Gualtiero I. Colombo

**Affiliations:** 1 Immunology and Functional Genomics Unit, Centro Cardiologico Monzino-IRCCS, 20138 Milan, Italy; mattia.chiesa@cardiologicomonzino.it (M.C.); martabuzztt@gmail.com (M.B.); vricci@ccfm.it (V.R.); 2 Vascular Biology and Regenerative Medicine Unit, Centro Cardiologico Monzino-IRCCS, 20138 Milan, Italy; carenamc86@gmail.com (M.C.C.); gpompilio@ccfm.it (G.P.); 3 Department of Medicine (DAME), University of Udine, 33100 Udine, Italy; antonio.beltrami@uniud.it (A.P.B.); ugolino.livi@uniud.it (U.L.); 4 Maria Cecilia Hospital, GVM Care & Research, 48033 Cotignola, Italy; rzzpla@unife.it (P.R.); roberto.ferrari@unife.it (R.F.); 5 Department of Morphology, Surgery and Experimental Medicine and Laboratory for Technologies of Advanced Therapies (LTTA), University of Ferrara, 44121 Ferrara, Italy; 6 Dipartimento di Medicina Clinica e Chirurgia, Università deli Studi di Napoli Federico II, 80131 Napoli, Italy; 7 Centro Cardiologico Universitario di Ferrara, University of Ferrara, 44124 Ferrara, Italy; a.fucili@ospfe.it; 8 Cardiovascular Department, Azienda Sanitaria Universitaria Giuliano Isontina (ASUGI), University of Trieste, 34148 Trieste, Italy; aaleksova@gmail.com; 9 Dipartimento di Scienze Cliniche e di Comunità, Università degli Studi di Milano, 20122 Milan, Italy

**Keywords:** microRNA, heart failure, biomarkers

## Abstract

(1)Background: Chronic heart failure (CHF) contributes to the overall burden of cardiovascular disease. Early identification of at-risk individuals may facilitate the targeting of precision therapies. Plasma microRNAs are promising circulating biomarkers for their implications with cardiac pathologies. In this pilot study, we investigate the possible exploitability of circulating micro-RNAs (miRNAs) to track chronic heart failure (CHF) occurrence, and progression from NYHA class I to IV. (2)Methods: We screened 367 microRNAs using TaqMan microRNA Arrays in plasma samples from healthy controls (HC) and CHF NYHA-class I-to-IV patients (5/group). Validation was performed by singleplex assays on 10 HC and 61 CHF subjects. Differences in the expression of validated microRNAs were evaluated through analysis of covariance (ANCOVA). Associations between N-terminal pro-BNP (NT-proBNP), left ventricular end-diastolic volume (LVEDV) or peak oxygen uptake (VO2 peak) and plasma microRNA were assessed by multivariable linear regression analysis. (3)Results: Twelve microRNAs showed higher expression in CHF patients vs. HC. Seven microRNAs were associated with NT-proBNP concentration; of these, miR-423-5p was also an independent predictor of LVEDV. Moreover, miR-499-5p was a predictor of the VO2 peak. Finally, a cluster of 5 miRNAs discriminated New York Heart Association (NYHA) class-I from HC subjects. (4)Conclusions: Our data suggest that circulating miRNAs have the potential to serve as pathophysiology-based markers of HF status and progression, and as indicators of pre-symptomatic individuals.

## 1. Introduction

Brain natriuretic peptide (BNP) and its non-active prohormone N-terminal pro-BNP (NT-proBNP) are the gold-standard circulating biomarkers (CBM) for heart failure (HF) [1], and are already included in current guidelines with Class I recommendation for diagnostic and prognostic purposes, as well as with Class II standing for assessing the risk of patient re-hospitalization, or for screening to prevent HF onset [2]. Both forms of HF, the form with reduced and the form with preserved ejection fraction (HFrEF and HFpEF), are well classified by natriuretic peptides (NPs), although HFrEF patients are those in which BNP and NT-proBNP show higher accuracy [3]. Nonetheless, although NPs represent the benchmark against which novel biomarkers must be compared [4], their circulating levels can be found altered in the presence of several conditions other than HF, including advancing age, renal failure, inflammatory cardiomyopathies, coronary heart disease, atrial arrhythmias, cardiotoxic drugs, and obesity [5,6].

Recent advances in the understanding of HF pathophysiology have better clarified the complex nature of HF as a heterogeneous group of syndromes, in which the role of CBM is deemed increasingly relevant not only to define disease state and rate but also to stand as a bridge between the different structural HF phenotypes and corresponding pharmacologic or device-based treatments [7]. The list of biologic processes involving HF has been enriched over time, including, so far, a plethora of pathologic states such as mitochondrial/metabolic abnormalities, neurohormonal activation, oxidative stress, extracellular matrix remodeling, inflammation, fibrosis, and apoptosis. The potential added value of novel pathophysiology-based HF-CBM on top of NPs might thus be recognized to result in a higher likelihood to identify, and possibly treat, specific subsets of patients according to the prevailing structural phenotype involved [8].

Interestingly, micro-RNAs (miRNAs), short (22-24 nt) non-coding RNAs, which are stably expressed in the systemic circulation of both animals and humans, have been introduced as potential pathophysiology-linked CBM [9]. Although the mechanisms underlying their release into the bloodstream are still poorly understood [10], circulating miRNAs have been proposed as diagnostic CBM in different pathologic conditions [11] (e.g., cancer, liver injury, hepatitis), including heart diseases [12]. We were among the first to report their potential as CBM in ST-elevated acute myocardial infarction [13] and in arrhythmogenic cardiomyopathy [14,15]. Similar findings have been described in other cardiac disease states, such as atrial fibrillation or coronary artery disease [16,17,18]. Notably, previous investigations have concordantly suggested that HF patients show dysregulation in the expression of several circulating miRNAs [19,20]. A recent comprehensive meta-analysis has failed, however, to confirm either single or pooled circulating miRNAs as diagnostic HF-CBM, except for promising data about miR-423-5p [21].

It is noteworthy that a major shortcoming of previous investigations focusing on HF-miRNAs relies upon the lack of comparative studies with NPs, which are instead warranted for better understanding the actual potential of miRNAs to gain a position in clinical practice. Another critical issue is the poor correlation of miRNAs’ blood levels with a thorough clinical characterization, including patients’ functional status and cardiac performance parameters.

In this study, we pave the way for large cohort-based studies by addressing the aforementioned issues in a highly homogeneous patient population well representative of chronic HF (CHF) progression along with NYHA classes and LV volumetric deterioration, to ascertain the ability of circulating miRNAs to unveil CHF onset and progression when compared to NPs.

## 2. Experimental Section

### 2.1. Patients

Sixty-one patients with HFrEF were enrolled by the outpatient clinic and hospital at Azienda Ospedaliero-Universitaria S. Anna. Diagnosis of ischemic and idiopathic dilated cardiomyopathy was based on accepted criteria [2]. Diagnosis of CHF was based on a history of HF for at least a six-month duration, reduced exercise tolerance, left ventricular functional impairment, and raised levels of NT-proBNP above the normal plasma level. Symptom-limited cardiopulmonary exercise testing was performed on a bicycle ergometer with a ramp protocol of 10 W/min. Exercise and gas-exchange data were collected continuously by using a computerized breath-by-breath gas-exchange analyzer. CHF clinical staging was performed according to the New York Heart Association (NYHA) classification. All patients received standard, evidence-based guided pharmacological treatment. The control group consisted of 10 healthy individuals, without any cardiovascular risk factor and receiving no treatment. Informed consent approved by the Regional Ethical Committee (code ef. 43/2009-211/2014/Em and Protocol 58635, October 2013) was obtained from all patients and controls, according to the World Medical Association Declaration of Helsinki. 

### 2.2. Natriuretic Peptide

NT-proBNP was evaluated by direct ELISA in EDTA plasma (DRG Instruments GmbH, Marburg, Germany). The minimum detection limit was 3 fmol/mL; the intra-assay (*n* = 16) and inter-assay (*n* = 10) coefficients of variation ranged from 5% to 8% and from 7% to 10%, respectively.

### 2.3. Total RNA Purification

Total RNA was extracted from plasma using TRIzol (Life Technologies, Carlsbad, CA, USA) following a modified protocol [13]. Briefly, 1 mL of TRIzol was added to each 400 µL of plasma, followed by the addition of 0.2 mL of chloroform and phase separation. After centrifugation, the upper aqueous phase was transferred to a new tube and 20 µg of glycogen (ThermoFisher Scientific, Carlsbad, CA, USA) and 1 mL of 100% isopropanol were added. Then, tubes were centrifuged again and the pellets washed with 70% ethanol, air-dried, resuspended in RNAse-free water, and stored at −80°C until use.

### 2.4. MicroRNA Screening

miRNA expression profiling was conducted using the TaqMan Human MicroRNA A Array v2.0 (Applied Biosystems, Carlsbad, CA, USA), following the manufacturer’s protocol. Reverse transcription and pre-amplification were conducted using Megaplex Primer Human Pools A v2.1 and TaqMan MicroRNA Reverse Transcription Kit. Data were analyzed using ExpressionSuite v1.1 software (Life Technologies, Carlsbad, CA, USA), using the Global Normalization method, and all miRNAs presenting Ct values > 35 in more than 50% of patients were considered as not expressed.

### 2.5. Single miRNA Assays

microRNA retro-transcription was conducted using TaqMan Advanced miRNA cDNA Synthesis Kit (Life Technologies, Carlsbad, CA, USA) starting from 2 µL of total RNA extract. Selected miRNAs were evaluated using single TaqMan Advanced miRNA assays (Life Technologies, Carlsbad, CA, USA), following the manufacturer’s protocol. Expression data were normalized using miR-532-5p as an internal reference not related to HF and presenting low variance throughout all study groups, experimentally validated as in previous works [13,14,18,22]. Normalized data were log2 transformed before statistical analysis.

### 2.6. Statistical Analysis

Categorical data were reported as counts and proportions, continuous data as mean ± SD. The normality of the data distribution was assessed by the D’Agostino-Pearson omnibus test. Categorical clinical variables were compared by the χ2 test or Fisher’s exact test. Between-group comparisons for continuous data were performed by one-way ANOVA or two-tailed unpaired Student’s *t*-test, as appropriate.

Data obtained from the miRNA screening were analyzed by the Kruskal-Wallis test, using the Benjamini-Hochberg correction for multiple testing. A False Discovery Rate (FDR) < 0.05 was considered statistically significant.

Differences in the expression of selected miRNA among the study groups were assessed by analysis of covariance (ANCOVA) using the general linear model approach. The models included miRNA expression values as dependent variables and NYHA class as the independent variable, and accounted for the effects of six factors, i.e., age, sex, diabetes mellitus, hypertension, hypercholesterolemia, and smoking habit. miRNAs with a *p*-value < 0.01 were considered statistically significant. Then, post-hoc multiple comparisons were made by Tukey’s test.

Relationships between parameters of HF severity were evaluated by Spearman’s coefficient of rank correlation (ρ) or Pearson correlation coefficient (r), when appropriate. Relationships between HF-severity variables and miRNA expression values were assessed by linear regression analysis adjusting for age, sex, personal history of diabetes mellitus, hypertension, hypercholesterolemia, and smoking habit, and corrected for multiple testing using the Benjamini-Hochberg procedure. Adjusted *p*-values < 0.05 were deemed statistically significant.

The mean decrease in the Gini index, calculated by an iterative Random Forests procedure implemented in the “randomForest” (www.rdocumentation.org, v4.6) R package, was used to rank the miRNAs based on their “importance” in discriminating controls vs. patients with early CHF stage. This score is a measure of how important a variable is for estimating the value of the target variable across all of the trees generated by a random forest classifier. The higher the mean decrease, the higher the variable importance. Top-ranked miRNA clustering analysis was performed by multi-dimensional scaling (MDS). The quality of clustering was evaluated by the average Silhouette Index (aSI) [23], taking into account the class membership, using the “cluster” (v2.0.7) R package; this index ranges from −1 to 1 and the higher the value, the better the discrimination power.

## 3. Results

### 3.1. Study Population Characteristics

All features of the study population are listed in Table 1. CHF patients enrolled in the study (*n* = 61) were distributed fairly evenly among the NYHA classes (class I *n* = 14, II *n* = 17, III *n* = 16, and IV *n* = 14) and most of them were males (67%). There were no substantial differences in risk factors and major drugs among patients in different NYHA classes. Mean left ventricular ejection fraction (LVEF) and peak oxygen uptake (VO2 peak) progressively and significantly declined in patients with worse NYHA functional class. In parallel, mean left ventricular end-diastolic volume (LVEDV) and NT-proBNP plasma concentration significantly increased as the NYHA class worsened. Finally, there were no significant differences in age and sex prevalence between healthy controls (HC, *n* = 10) and CHF patients.

### 3.2. Correlation between NYHA Class, LVEDV, and NT-proBNP Levels

**Figure 1:**
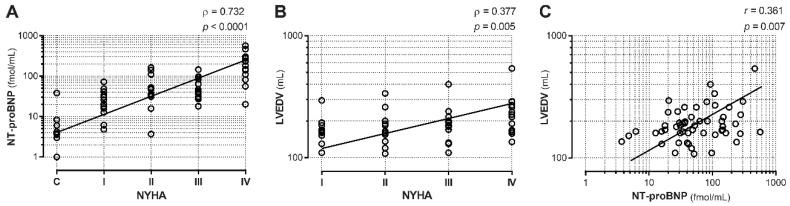
Correlation between heart failure severity indexes. Two markers of chronic heart failure (CHF) severity, NT-proBNP, and LVEDV, were analyzed for their correlation with NYHA classes. (**A**) NT-proBNP levels showed a positive correlation with disease stage (C, healthy controls, and I to IV, NYHA class). (**B**) LVEDV also showed a positive correlation with HF NYHA classes. (**C**) LVEDV showed a positive correlation with NT-proBNP levels in NYHA patients. Trendlines are depicted. C *n* = 10, NYHA I *n* = 14, NYHA II *n* = 17, NYHA III *n* = 16, NYHA IV *n* = 14.

To confirm that NYHA class progression was paralleled by increases in NT-proBNP and LVEDV, we evaluated the correlations between NYHA class and these two HF severity indexes. Our analysis showed highly positive correlations between NYHA class and NT-proBNP (Figure 1A; ρ = 0.732, *p* < 0.001) or LVEDV (Figure 1B; ρ = 0.377, *p* < 0.005), indicating a proper CHF patient allocation for disease severity and the expected relationships between functional status, NP levels, and LV remodeling. Indeed, there was a significant correlation between LVEDV and NT-proBNP (Figure 1C; r = 0.361, *p* < 0.01).

### 3.3. Circulating miRNAs Expression and in Relation with NYHA Class

**Figure 2:**
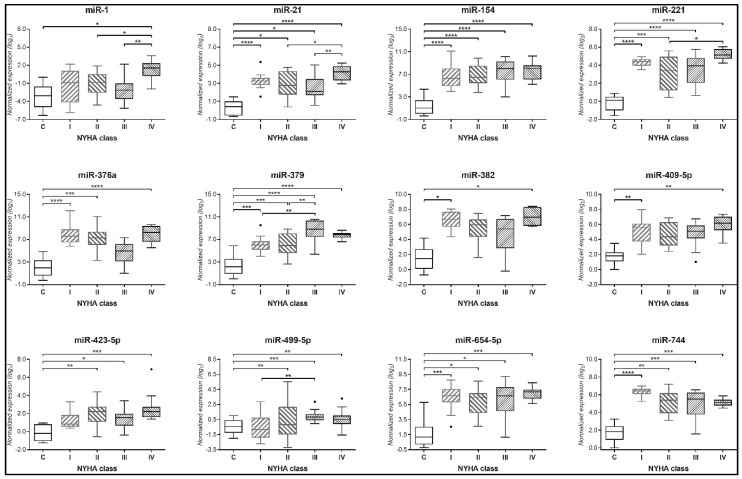
Plasma miRNAs regulated in Heart Failure. Twelve circulating miRNAs showed increased levels in patients belonging to NYHA class I to IV vs. healthy controls (C), even after adjustment for age, sex, personal history of diabetes mellitus, hypertension, and/or hypercholesterolemia, and smoking habit. These miRNAs were all significant at the analysis of covariance (ANCOVA) for *p* < 0.01. The expression of each miRNA is represented by box and whiskers plots. C *n* = 10, NYHA I *n* = 14, NYHA II *n* = 17, NYHA III *n* = 16, NYHA IV *n* = 14. * *p* < 0.05, ** *p* < 0.01, *** *p* < 0.001, **** *p* < 0.0001 at Tukey’s multiple comparison test.

We conducted a miRNA profiling on plasma samples obtained from patients of the four NYHA classes and HC (5/group). The screening revealed 234 detectable miRNAs (Table S1). Several miRNAs showed a putative differential expression when comparing CHF patients vs. HC (data not shown). For the validation step on all samples, we selected a restricted group of 17 candidate miRNAs showing both high regulation and statistical significance. By singleplex assays and ANCOVA analysis (Table S2), we found that 12 circulating miRNAs showed significantly higher expression in most CHF NYHA classes than in the HC group: i.e., miR-1, -154, -21, -221, -376a, -379, -382, -409-5p, -423-5p, -499-5p, -654-5p, and -744 (Figure 2). Of note, miR-423-5p and -499-5p were elevated in NYHA classes II-to-IV, but not in class I, compared to HC. All other miRNAs, except miR-1, were found with a higher expression in NYHA class I patients and at least one other NYHA class compared to HC. Only miR-379 expression levels showed a trend that mirrored the progression of CHF through class I to III, reaching a plateau at classes III and IV.

### 3.4. Correlation between Plasma miRNA Levels and NT-proBNP, LVEDV, and VO2 Peak

**Figure 3:**
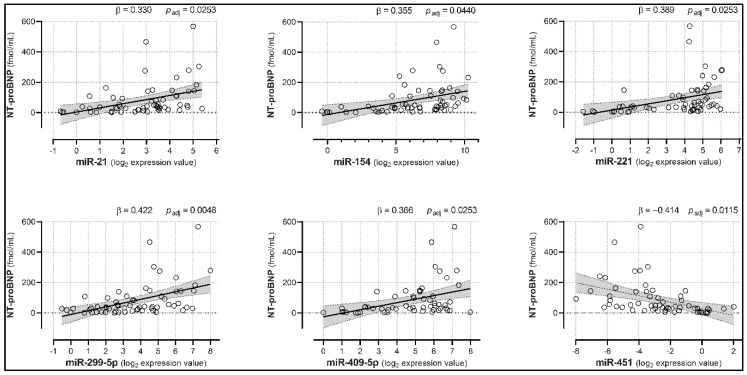
Circulating miRNAs associated with NT-proBNP plasma concentration. Five miRNAs showed positive associations, whereas one miRNA was negatively associated with NT-proBNP levels. Analyses were adjusted for age, sex, diabetes mellitus, hypertension, hypercholesterolemia, and smoking habit. Trendlines are depicted along with the 95% confidence interval (light gray). *p_adj_* = *p*-values corrected for multiple testing; *n* = 61.

**Figure 4:**
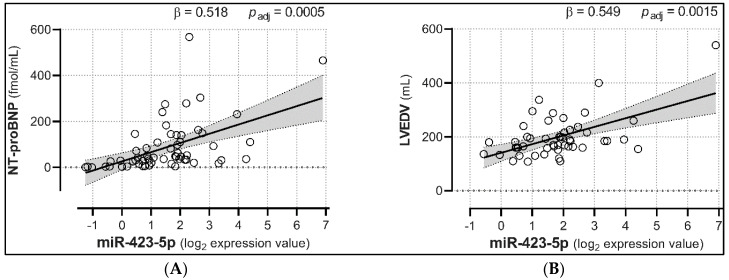
Expression levels of plasma miR-423-5p are associated with both NT-proBNP and LVEDV. HF-regulated miR-423-5p showed positive associations with both NT-proBNP (**A**) and LVEDV (**B**). Analyses were adjusted for age, sex, personal history of diabetes mellitus, hypertension, and/or hypercholesterolemia, and smoking habit. Trendlines are depicted along with the 95% confidence interval (light gray). *p_adj_* = *p*-values corrected for multiple comparisons; *n* = 61.

**Figure 5:**
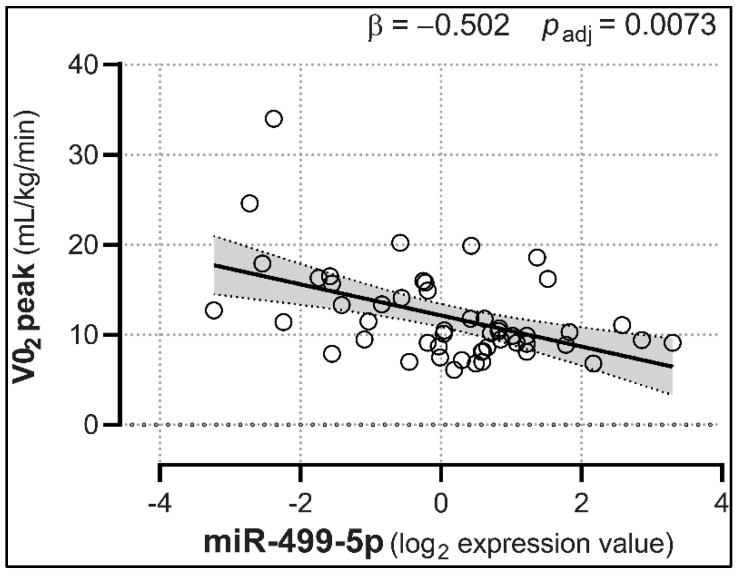
Expression levels of plasma miR-499-5p are associated with the VO_2_ peak. HF-regulated miR-499-5p showed a negative association with VO_2_ peak levels. Analyses were adjusted for age, sex, personal history of diabetes mellitus, hypertension, and/or hypercholesterolemia, and smoking habit. Trendlines are depicted along with the 95% confidence interval (light gray). *p_adj_* = *p*-values corrected for multiple comparisons; *n* = 61.

We then investigated the relationships between miRNA expression and NT-proBNP levels in HC and NYHA subjects, as well as LVEDV and VO2 peak changes in NYHA patients. Linear regression analyses showed that miR-21, -154, -221, -299-5p, and -409-5p were positively associated with NT-proBNP levels (with β coefficients ranging from 0.330 to 0.422, adjusted *p* < 0.05; Figure 3 and Table 2), suggesting that their increase may well predict the progressive rise in NPs. Conversely, miR-451 was negatively correlated with NT-proBNP (β = −0.414, adjusted *p* = 0.0115). Furthermore, and most importantly, miR-423-5p showed significant positive associations with both NT-proBNP (β = 0.518, adjusted *p* = 0.0005), and LVEDV (β = 0.549, adjusted *p* = 0.0015; Figure 4), hinting at its potential ability to track both HF severity and LV remodeling. No other miRNA was a predictor of LVEDV levels (Table S3). Lastly, miR-499-5p was the only miRNA presenting a (negative) association with VO2 peak levels (β = −0.502, adjusted *p* = 0.0073; Figure 5 and Table S4).

### 3.5. Identification of a miRNA Signature of Early-Stage CHF

**Figure 6:**
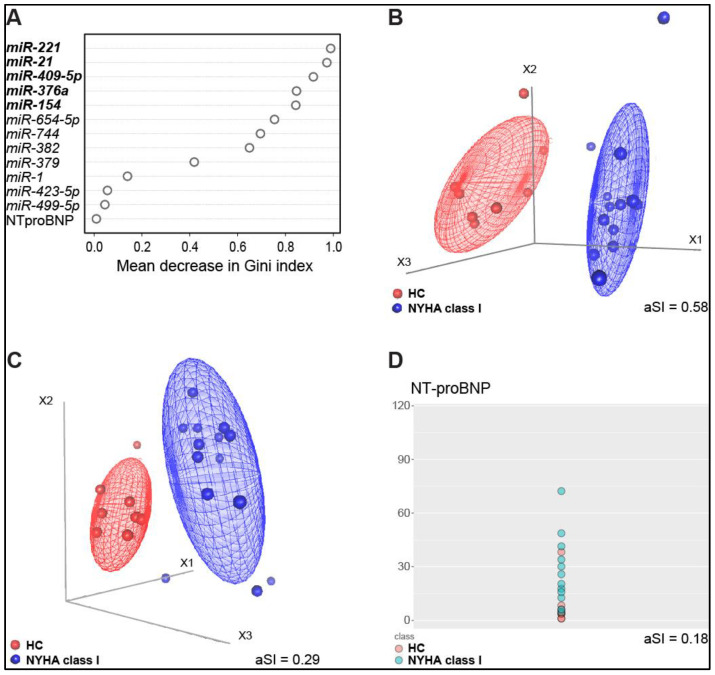
Discriminating potential of miRNAs and NT-proBNP when comparing HC and NYHA I class subjects. (**A**) Variables ranking based on the mean decrease in the Gini index classifying healthy controls HC vs. NYHA class I samples. (**B**) 3D scatterplot based on miR-221, -21, -409-5p, -376a, and -154, shows a complete separation between HC and NYHA I class subjects. (**C**) 3D scatterplot depicting the decrease in discrimination power when NT-proBNP was added to the miRNA signature. (**D**) Scatterplot showing the performance of NT-proBNP in distinguishing HC from NYHA class I patients. (**A**–**D**): HC: *n* = 10; NYHA class I samples: *n* = 14. (**B**,**C**): Red dots: healthy controls. Blue dots NYHA class I patients. aSI, average Silhouette Index.

We finally sought to identify which miRNAs had the highest ability to discriminate between HC and early HF stage, as defined by NYHA class I, comparing their “performance” with NT-proBNP standard measurement. As shown in Figure 6A, miR-221 had the highest mean decrease in the Gini index, which means that this miRNA was the most informative variable to distinguish HC from NYHA class I. Furthermore, exploiting MDS cluster analysis, we found that the top 5 miRNAs in the Gini index ranking (i.e., miR-221, -21, -409-5p, -376a, and -154) could reach the highest discrimination power, as assessed by the average Silhouette Index (aSI=0.58, Figure 6B). For comparison, we assessed the performance of this miRNA cluster on top of NT-proBNP (Figure 6C). Indeed, when NT-proBNP was added to the set of 5 miRNAs, the aSI dropped to 0.29, lowering the discrimination capacity of the miRNA signature. NT-proBNP alone showed the worst performance, with an aSI of 0.18 (Figure 6D).

## 4. Discussion

The main findings of this study are: (i) a cluster of 12 circulating miRNAs that were found dysregulated in a well-characterized CHF population split along NYHA classes; (ii) a group of 6 miRNAs (miR-21, -154, -221, -299-5p, -409-5p, and -423-5p) that well-matched NT-proBNP rise along NYHA classes, with miR-423-5p also accurately tracking LV-adverse dilation; (iii) one miRNA (miR-499-5p), that was able to predict the declining capacity of oxygen uptake as the NYHA class worsens; (iv) a signature composed of miR-221, -21, -409-5p, -376a, and -154 that well discriminate early-stage asymptomatic CHF patients (NYHA class I) from healthy controls. We believe this work integrates previous information about the capability of circulating miRNAs to detect CHF onset and monitor its progression on top of NPs, providing at the same time novel perspectives in the field of circulating miRNAs as promising biomarkers in heart failure for diagnostic purposes.

To better embrace the spectral nature of CHF, an emerging need for more personalized and mechanistic approaches has been proposed, in which clinical studies with smaller but more homogeneous patient populations are required [24]. We have taken advantage of stringent and state-of-the-art criteria of patient selection based on clinical history, functional class, and LV performance as well as NP circulating levels to achieve a relatively small but homogeneous patient sample that represents HF status along with NYHA classes and progressive LV adverse dilation. The check for a positive correlation between NYHA class, LVEDV progression, and NT-proBNP levels reassured us about our ability to properly target such an accurately identified CHF population.

There is currently emerging consensus that the search for reliable biomarkers is justified on the basis of the evidence that CHF is a mixture of different structural phenotypes [8] that need to be addressed by a novel approach considering CBM as a bridge between a given phenotype and potential treatment strategies. In this regard, plasma miRNAs can be theoretically viewed as good CBM candidates in CHF.

Nevertheless, a recent meta-analysis taking into account 10 prospective studies evaluating miRNAs’ performance in CHF diagnosis has identified only miR-423-5p with the potential to be a biomarker for CHF diagnosis [21]. This result is surprising, considering the high number of candidate miRNAs (>30). There is, however, considerable heterogeneity among studies, in terms of assessment of golden standard criteria (only 1 study has matched clinical, echocardiographic, and NP data) as well as in the comparison of miRNAs expression with both BNP levels and clinical profile (2 studies only have evaluated an NYHA class).

The present work was aimed at evaluating miRNAs’ exploitability as diagnostic and staging CBM for CHF, and we believe that the main emerging findings are worth scrutiny. First, among 12 circulating miRNAs found increased in patients with CHF, a small cluster composed of miR-1, -423-5p, and -499-5p was found to well match the symptomatic stage of the disease (from NYHA class II to IV). All of these 3 miRNAs have been previously described as deeply involved in cardiac pathophysiology. Both miR-1 and -499-5p are involved in processes typical of HF: the former regulates hypertrophy [25], and the latter is associated with cell senescence and terminal differentiation [26]. Finally, miRNA-423-5p correlated with BNP levels and was found increased in patients with systolic HF, as well as in dilated cardiomyopathy [27,28]. Even more interesting, miR-423-5p showed a significant positive correlation with both NT-proBNP and LVEDV. To the best of our knowledge, this is the first time that the potential of miRNAs to simultaneously track both HF severity and LV remodeling trend has been reported. It is worth noting that cardiac injury has been shown to increase the expression of miR-423-5p in cardiomyocytes in a time- and concentration-dependent manner and that its silencing significantly protected cardiomyocytes from apoptosis [29].

Of note, we found that miR-499-5p expression was higher in NYHA class II-to-IV HF patients and, at the same time, negatively associated with VO_2_ peak levels. To our knowledge, this is a novel observation, suggesting a possible role for miR-499-5p as a marker of reduced peak aerobic power and exercise capacity.

Finally, we found that a cluster composed of 5 miRNAs (miR-221, -21, -409-5p, -376a, and -154) could reach the highest discrimination power between healthy control subjects and early CHF stage (NYHA class I) when compared to NT-proBNP. This is another interesting unprecedented finding, given the undisputed need to recognize LV-dysfunction patients in the early asymptomatic stage. Four of these miRNAs are known to be associated with cardiovascular diseases. In particular, miRNA-221 was associated with left ventricular stiffness in pressure-overload HF and myocardial fibrosis [30], similar to miRNA-21 [31]. The latter was also found to be linked to pressure-overload aortic stenosis, correlating with collagen expression in the heart [32]. The expression of miR-376 was found to be associated with myocardial injury in a pig model of coronary microembolization [33]. Finally, miR-154 plays a key role in activating cardiac fibroblasts’ transdifferentiation into myofibroblasts [34]. MiR-409 was never associated with heart conditions but was reported to induce epithelial-to-mesenchymal transition in tumor-stromal interaction [35].

The main limitation of this work is the small patient sample size. Thus, it should be considered as a pilot study to pave the way for further large cohort-based investigations. However, we believe that the stringent selection criteria applied for patient selection throughout the full spectrum of NYHA classes may partially overcome such shortcomings. Our miRNA screening was conducted on a limited number of miRNAs, namely those that were present on the commercially available array. Despite not being a genome-wide scan, the sensitivity and accuracy of the TaqMan assays allowed us to evaluate the expression of a very consistent number of plasma miRNAs.

## 5. Conclusions

In conclusion, this is the first work showing the potential of circulating miRNAs as both markers of CHF presence and progression and signs of CHF onset in the absence of a pathological phenotype, as in the case of NYHA class I patients. Further investigations are warranted to confirm these results and to improve and translate the miRNA signatures identified to the clinical setting. This work highlights the potential of circulating miRNAs as markers of the presence and progression of HF. Interestingly, miRNAs’ signatures identified here show biologic implications with the multicomponent HF pathogenesis, onset, and progression. The added value on top of NPs of these markers relies upon a more precise identification of HF phenotypes, which may allow to more accurately design personalized therapies. Previous works showed that circulating miRNAs can be useful indicators of response to treatments aimed at ameliorating the cardiac function of HF patients [36,37]. If confirmed by further clinical investigations, the miRNA signatures identified in this work may be adopted as pathophysiology-based markers in the clinical setting. In particular, their ability to work as indicators of a pre-symptomatic HF stage (NYHA class I patients) could be translated into the early treatment of a disease that has a tremendous impact on patients’ life quality and expectancy.

## Figures and Tables

**Table 1:** Demographic and clinical features of the study population.

Characteristic	NYHA I (*n* = 14)	NYHA II (*n* = 17)	NYHA III (*n* = 16)	NYHA IV (*n* = 14)	*p*-Value	HC (*n* = 10)	*p*-Value
Age, years	64 ± 6.4	70.8 ± 10.4	69.2 ± 10.4	68.4 ± 8.6	0.23	62.7 ± 2.3	0.08
Male sex, n (%)	11 (79%)	10 (59%)	11 (69%)	9 (64%)	0.71	9 (90%)	0.47
NT-proBNP, fmol/mL	27.5 ± 19.5	66.2 ± 51.4	60.3 ± 41	221.6 ± 153.3	<0.001	18.3 ± 35.9	<0.001
Current smoker, n (%)	8 (57%)	8 (47%)	11 (69%)	8 (57%)	0.72	0 (0%)	
CAD familiarity, n (%)	6 (43%)	9 (53%)	8 (50%)	5 (36%)	0.8	0 (0%)	
Ischemic aetiology, n (%)	10 (71%)	10 (63%)	12 (75%)	9 (64%)	0.9	0 (0%)	
Hypertension, n (%)	7 (50%)	10 (59%)	12 (75%)	5 (36%)	0.18	0 (0%)	
Hypercholesterolemia, n (%)	11 (79%)	12 (71%)	11 (69%)	5 (36%)	0.1	0 (0%)	
Diabetes, n (%)	4 (29%)	4 (24%)	5 (31%)	8 (57%)	0.26	0 (0%)	
LVEDV, mL	163.2 ± 45.2	174.9 ± 58.7	189.9 ± 65.9	242.9 ± 99.4	0.02	NA	
LVEDD, mm	59.3 ± 5.3	61.9 ± 8.8	62.3 ± 9.2	64.8 ± 8.8	0.4	NA	
LVEF, %	38.2 ± 4.4	35.6 ± 8.6	32.1 ± 7.4	24.4 ± 7.3	<0.001	NA	
PAP, mmHg	31.2 ± 5.8	35.9 ± 15.6	32.4 ± 5.7	42.1 ± 13.2	0.23	NA	
VO2 peak, mL/kg/min	16.4 ± 6.9	15.2 ± 3.3	9.9 ± 0.9	7.9 ± 1	<0.001	NA	
ACEIs, n (%)	11 (79%)	12 (71%)	12 (75%)	7 (50%)	0.37	0 (0%)	
ARAs, n (%)	3 (25%)	6 (50%)	4 (25%)	8 (57%)	0.2	0 (0%)	
ARBs, n (%)	9 (%)	12 (53%)	12 (69%)	7 (43%)	0.54	0 (0%)	
Beta blockers, n (%)	13 (93%)	16 (94%)	13 (81%)	13 (93%)	0.67	0 (0%)	
Calcium antagonists, n (%)	3 (21%)	1 (6%)	1 (6%)	0 (0%)	0.25	0 (0%)	
Digitalis, n (%)	2 (14%)	4 (24%)	7 (44%)	5 (36%)	0.32	0 (0%)	
Diuretics, n (%)	9 (64%)	15 (88%)	14 (88%)	14 (100%)	0.06	0 (0%)	
Nitrates, n (%)	2 (14%)	3 (17%)	6 (38%)	2 (14%)	0.39	0 (0%)	
PUFAs, n (%)	9 (64%)	7 (41%)	7 (44%)	5 (36%)	0.48	0 (0%)	
Statins, n (%)	8 (57%)	9 (53%)	11 (69%)	6 (43%)	0.59	0 (0%)	

Values are expressed as mean ± SD for continuous variables and count and percentage (round brackets) for categorical variables. NYHA, New York Heart Association; HC, healthy control; NT-proBNP, N-terminal pro-brain natriuretic peptide; CAD, coronary artery disease; LVEDV, left ventricular end-diastolic volume; LVEDD, left ventricular end-diastolic diameter; LVEF left ventricular ejection fraction; PAP, pulmonary artery pressure; VO2 peak, peak oxygen uptake; NA, not available; ACEIs, angiotensin-converting-enzyme inhibitors; ARAs, aldosterone receptor antagonists; ARBs, angiotensin II receptor blockers; PUFAs, polyunsaturated fatty acids.

**Table 2:** Associations between microRNAs and NT-proBNP concentration.

microRNA	B	SE	95% CI for B	β	95% CI for β	*p*-Value	*p*_adj_
Mir-1	11.810	5.35	1.089, 22.53	0.264	0.024, 0.504	0.03149	0.05353
Mir-124a	−3.040	5.22	−13.508, 7.44	−0.073	−0.325, 0.179	0.56355	0.59635
Mir-154	14.120	5.80	2.504, 25.74	0.355	0.063, 0.646	0.01813	0.04403
Mir-21	22.630	8.31	5.96, 39.29	0.330	0.087, 0.572	0.00874	0.02533
Mir-221	20.732	7.54	5.61, 35.85	0.389	0.105, 0.672	0.00815	0.02533
Mir-299-5p	21.906	5.97	9.934, 33.88	0.422	0.191, 0.652	0.00056	0.00476
Mir-331-5p	−5.860	11.00	−27.949, 16.23	−0.080	−0.381, 0.221	0.59635	0.59635
Mir-375	−17.890	8.04	−34.003, −1.77	−0.261	−0.496, −0.026	0.03025	0.05353
Mir-376a	10.810	4.78	1.216, 20.4	0.284	0.032, 0.536	0.02796	0.05353
Mir-379	12.700	6.09	0.49, 24.91	0.295	0.011, 0.578	0.04178	0.05464
Mir-382	11.750	5.45	0.757, 22.74	0.387	0.025, 0.748	0.03676	0.05464
Mir-409-5p	20.970	7.70	5.492, 36.44	0.366	0.096, 0.636	0.00894	0.02533
Mir-423-5p	40.190	8.69	22.74, 57.63	0.518	0.293, 0.743	0.00003	0.00051
Mir-451	−20.150	6.20	−32.59, −7.71	−0.414	−0.669, −0.158	0.00203	0.01150
Mir-499-5p	5.520	9.62	−13.788, 24.82	0.070	−0.176, 0.316	0.56890	0.59635
Mir-654-5p	15.130	7.17	0.759, 29.5	0.318	0.016, 0.621	0.03945	0.05464
Mir-744	6.140	7.25	−8.504, 20.78	0.148	−0.205, 0.501	0.40244	0.48868

Analyses were adjusted for age, sex, personal history of diabetes mellitus, hypertension, hypercholesterolemia, and smoking habit. B = unstandardized regression coefficient; SE = standard error; 95% CI = 95% confidence interval of regression coefficients; β = standardized regression coefficient; *p*_adj_ = Benjamini-Hochberg adjusted *p*-value.

## Supplementary Materials

The following are available online at https://www.mdpi.com/2227-9059/8/12/597/s1, Table S1: Expression data of miRNA Screening. Table S2: Results of the ANCOVA analysis for the class membership (healthy and NYHA class I to IV). Table S3: Linear regression analysis of microRNA associations with LVEDV, Table S4: Linear regression analysis of microRNA associations with VO2 peak.
