# Supplementary material for: Differential Role of Circulating microRNAs to Track Progression and Pre-Symptomatic Stage of Chronic Heart Failure: A Pilot Study
Source: Biomedicines. 2020 Dec 11;8(12):597. doi: 10.3390/biomedicines8120597 (PMC7764340; doi:10.3390/biomedicines8120597)
Supplement: Supplementary file 1 [file biomedicines-08-00597-s001.zip › DAlessandra et al Supplementary Table S2.docx]

**Table S2. Results of the ANCOVA analysis for the class membership (healthy and NYHA class I to IV)**

| **microRNA** | **F** | ***P*-value** |
| --- | --- | --- |
| miR-1 | 6.713 | **0.00016** |
| miR-124a | 1.501 | 0.21349 |
| miR-154 | 9.911 | **< 0.00001** |
| miR-21 | 13.580 | **< 0.00001** |
| miR-221 | 16.455 | **< 0.00001** |
| miR-299-5p | 2.936 | 0.02802 |
| miR-331-5p | 2.162 | 0.08569 |
| miR-375 | 3.086 | 0.02236 |
| miR-376a | 15.594 | **< 0.00001** |
| miR-379 | 12.604 | **< 0.00001** |
| miR-382 | 5.405 | **0.00137** |
| miR-409-5p | 7.030 | **0.00013** |
| miR-423-5p | 7.013 | **0.00012** |
| miR-451 | 2.701 | 0.03975 |
| miR-499-5p | 3.781 | **0.00853** |
| miR-654-5p | 7.213 | **0.00009** |
| miR-744 | 13.098 | **< 0.00001** |

Analyses were corrected for age, sex, personal history of diabetes mellitus, hypertension, and/or hypercholesterolemia, and smoking habit. miRNAs with a P-value <0.01 were deemed as statistically significant. Post-hoc pairwise comparisons were performed on these miRNAs using the Tukey's test (*cf.* Figure 2).
