# Supplementary material for: Differential Role of Circulating microRNAs to Track Progression and Pre-Symptomatic Stage of Chronic Heart Failure: A Pilot Study
Source: Biomedicines. 2020 Dec 11;8(12):597. doi: 10.3390/biomedicines8120597 (PMC7764340; doi:10.3390/biomedicines8120597)
Supplement: Supplementary file 1 [file biomedicines-08-00597-s001.zip › DAlessandra et al Supplementary table S3.docx]

**Table S3. Linear regression analysis of microRNA associations with LVEDV**

| **microRNA** | **B** | | | **SE** | **95% CI for B** | | **β** | | **95% CI for β** | | ***P*-value** | | ***P*_adj_** |
| --- | --- | --- | --- | --- | --- | --- | --- | --- | --- | --- | --- | --- | --- |
| miR_1 | | -2.677 | 5.12 | | -13.00, 7.64 | -0.080 | | -0.387, 0.228 | | 0.60376 | | 0.74599 | |
| miR_124a | | 7.125 | 3.96 | | -0.85, 15.10 | 0.252 | | -0.030, 0.534 | | 0.07871 | | 0.43031 | |
| miR_154 | | 6.017 | 6.04 | | -6.14, 18.17 | 0.147 | | -0.150, 0.445 | | 0.32433 | | 0.74599 | |
| miR_21 | | -9.629 | 9.10 | | -27.96, 8.71 | -0.155 | | -0.451, 0.141 | | 0.29565 | | 0.74599 | |
| miR_221 | | -7.005 | 8.46 | | -24.06, 10.05 | -0.137 | | -0.471, 0.197 | | 0.41204 | | 0.74599 | |
| miR_299_5p | | 2.245 | 5.34 | | -8.50, 12.99 | 0.061 | | -0.229, 0.350 | | 0.67596 | | 0.74599 | |
| miR_331_5p | | 3.198 | 8.31 | | -13.55, 19.95 | 0.061 | | -0.257, 0.378 | | 0.70211 | | 0.74599 | |
| miR_375 | | 2.959 | 6.69 | | -10.51, 16.43 | 0.063 | | -0.223, 0.348 | | 0.66043 | | 0.74599 | |
| miR_376a | | -1.720 | 4.12 | | -10.03, 6.59 | -0.058 | | -0.340, 0.224 | | 0.67866 | | 0.74599 | |
| miR_379 | | 3.840 | 5.94 | | -8.11, 15.80 | 0.097 | | -0.204, 0.398 | | 0.52066 | | 0.74599 | |
| miR_382 | | 6.589 | 7.24 | | -8.13, 21.31 | 0.165 | | -0.203, 0.533 | | 0.36944 | | 0.74599 | |
| miR_409_5p | | -7.375 | 7.87 | | -23.28, 8.53 | -0.145 | | -0.457, 0.168 | | 0.35429 | | 0.74599 | |
| miR_423_5p | | 32.090 | 7.39 | | 17.18, 46.995 | 0.549 | | 0.294, 0.804 | | **0.00009** | | **0.00153** | |
| miR_451 | | -8.981 | 5.02 | | -19.08, 1.12 | -0.257 | | -0.546, 0.032 | | 0.08008 | | 0.43031 | |
| miR_499_5p | | -5.756 | 7.55 | | -20.98, 9.47 | -0.108 | | -0.392, 0.177 | | 0.44995 | | 0.74599 | |
| miR_654_5p | | 12.170 | 7.27 | | -2.48, 26.83 | 0.256 | | -0.052, 0.563 | | 0.10125 | | 0.43031 | |
| miR_744 | | 1.898 | 11.11 | | -20.70, 24.50 | 0.029 | | -0.318, 0.376 | | 0.86538 | | 0.86538 | |

Analyses were adjusted for age, sex, personal history of diabetes mellitus, hypertension, hypercholesterolemia, and smoking habit. LVEDV = left ventricular end-diastolic volume; B = unstandardized regression coefficient; SE = standard error; 95% CI = 95% confidence interval of regression coefficients; β = standardized regression coefficient; *P*_adj_ = Benjamini-Hochberg adjusted *P*-value.
