# Supplementary material for: Differential Role of Circulating microRNAs to Track Progression and Pre-Symptomatic Stage of Chronic Heart Failure: A Pilot Study
Source: Biomedicines. 2020 Dec 11;8(12):597. doi: 10.3390/biomedicines8120597 (PMC7764340; doi:10.3390/biomedicines8120597)
Supplement: Supplementary file 1 [file biomedicines-08-00597-s001.zip › DAlessandra et al Supplementary Table S4.docx]

**Table S4. Linear regression analysis of microRNA associations with VO_2_ peak**

| **microRNA** | **B** | | | **SE** | | **95% CI for B** | | **β** | | **95% CI for β** | | ***P*-value** | | ***P*_adj_** |
| --- | --- | --- | --- | --- | --- | --- | --- | --- | --- | --- | --- | --- | --- | --- |
| miR_1 | | 0.149 | 0.37 | | -0.591, 0.889 | | 0.066 | | -0.264, 0.396 | | 0.68712 | | 0.98654 | |
| miR_124a | | -0.509 | 0.28 | | -1.080, 0.063 | | -0.269 | | -0.571, 0.033 | | 0.07986 | | 0.39551 | |
| miR_154 | | -0.347 | 0.43 | | -1.220, 0.527 | | -0.127 | | -0.447, 0.193 | | 0.42822 | | 0.98654 | |
| miR_21 | | 0.058 | 0.66 | | -1.271, 1.388 | | 0.014 | | -0.307, 0.335 | | 0.92995 | | 0.98654 | |
| miR_221 | | -0.032 | 0.60 | | -1.239, 1.174 | | -0.010 | | -0.369, 0.350 | | 0.95748 | | 0.98654 | |
| miR_299_5p | | -0.092 | 0.38 | | -0.863, 0.679 | | -0.037 | | -0.347, 0.273 | | 0.81151 | | 0.98654 | |
| miR_331_5p | | 0.394 | 0.60 | | -0.807, 1.594 | | 0.112 | | -0.230, 0.454 | | 0.51178 | | 0.98654 | |
| miR_375 | | -0.008 | 0.48 | | -0.974, 0.958 | | -0.003 | | -0.309, 0.304 | | 0.98654 | | 0.98654 | |
| miR_376a | | 0.126 | 0.39 | | -0.650, 0.902 | | 0.050 | | -0.260, 0.361 | | 0.74518 | | 0.98654 | |
| miR_379 | | -0.842 | 0.41 | | -1.663, -0.021 | | -0.318 | | -0.627, -0.008 | | **0.04457** | | 0.37885 | |
| miR_382 | | 0.232 | 0.49 | | -0.772, 1.236 | | 0.088 | | -0.292, 0.468 | | 0.64173 | | 0.98654 | |
| miR_409_5p | | 0.018 | 0.56 | | -1.104, 1.141 | | 0.005 | | -0.330, 0.341 | | 0.97399 | | 0.98654 | |
| miR_423_5p | | -1.059 | 0.62 | | -2.302, 0.185 | | -0.272 | | -0.591, 0.047 | | 0.09306 | | 0.39551 | |
| miR_451 | | 0.056 | 0.37 | | -0.691, 0.804 | | 0.024 | | -0.296, 0.344 | | 0.88046 | | 0.98654 | |
| miR_499_5p | | -1.793 | 0.47 | | -2.741, -0.844 | | -0.502 | | -0.767, -0.236 | | **0.00043** | | **0.00731** | |
| miR_654_5p | | -0.026 | 0.54 | | -1.105, 1.054 | | -0.008 | | -0.346, 0.330 | | 0.96202 | | 0.98654 | |
| miR_744 | | 1.112 | 0.73 | | -0.366, 2.591 | | 0.258 | | -0.085, 0.602 | | 0.13541 | | 0.46039 | |

Analyses were adjusted for age, sex, personal history of diabetes mellitus, hypertension, hypercholesterolemia, and smoking habit. VO_2_ peak = peak oxygen uptake; B = unstandardized regression coefficient; SE = standard error; 95% CI = 95% confidence interval of regression coefficients; β = standardized regression coefficient; *P*_adj_ = Benjamini-Hochberg adjusted *P*-value.
